# Impact of Pulsed Field Ablation Dosing on Outcome After Pulmonary Vein Isolation Using a Pentaspline Ablation Catheter: A Prospective Comparison of 8 Versus 16 Applications

**DOI:** 10.1111/jce.70266

**Published:** 2026-01-20

**Authors:** Katharina Ji‐Mi Yang, Maximilian Spieker, Stephan Angendohr, Carsten auf der Heiden, David Glöckner, Roberto Sansone, Malte Kelm, Alexandru Gabriel Bejinariu, Obaida Rana

**Affiliations:** ^1^ Division of Cardiology, Pulmonology and Vascular Medicine, Medical Faculty University Hospital Duesseldorf, Heinrich‐Heine University Duesseldorf Duesseldorf Nordrhein‐Westfalen Germany; ^2^ Cardiovascular Research Institute Duesseldorf, Medical Faculty Heinrich‐Heine University Duesseldorf Germany

**Keywords:** atrial fibrillation, dosing, PFA, pulmonary vein isolation

## Abstract

**Background:**

Pulsed field ablation (PFA) is a novel, non‐thermal ablation modality to achieve pulmonary vein isolation (PVI) for the treatment of atrial fibrillation (AF). For the pentaspline catheter, eight applications per pulmonary vein (PV) are considered standard, with four in basket configuration and four in flower configuration. The objective of the study is to investigate whether pentaspline PFA using 16 applications per PV is associated with an improved outcome compared with the standard procedure.

**Methods:**

The prospective study included a total of 292 patients with AF. According to the number of applications per PV, patients were assigned to group PFA‐8 (8 applications per PV; 4x basket configuration, 4x flower configuration; *n* = 130) or group PFA‐16 (16 applications per PV; 6x basket, 10x flower; *n* = 162). The primary endpoint was freedom from atrial arrhythmia (AA), i.e. AF, atrial flutter, and atrial tachycardia, after a follow‐up period of 1 year, as assessed by Holter monitoring after 3 and 12 months, respectively.

**Results:**

Freedom from AA was significantly higher in group PFA‐16 than in group PFA‐8 (73.9% vs. 62.3%; *p* < 0.05). Subgroup analysis showed greater effectiveness in the PFA‐16 group than in the PFA‐8 group (66.1% vs. 45.3%; *p* < 0.05) in patients with persistent AF, while freedom from AA was similar in both groups in patients with paroxysmal AF (78.8% in PFA‐16 vs. 75.7% in PFA‐8; *p *= ns). Serious adverse events were observed in 8 (2.7%) patients, with no differences between the two groups.

**Conclusion:**

PVI using 16 PFA applications per PV may improve clinical outcome in patients with persistent AF.

## Graphical abstract

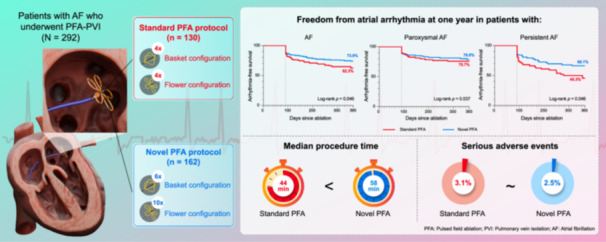

**Abbreviations**
AA: atrial arrhythmia
AAD: antiarrhythmic drugs
AF: atrial fibrillation
AKI: acute kidney injury
CBA: cryoballoon ablation
eGFR: estimated glomerular filtration rate
PAF: paroxysmal atrial fibrillation
PersAF: persistent atrial fibrillation
PFA: pulsed field ablation
PV: pulmonary vein
PVI: pulmonary vein isolation
RFA: radiofrequency ablation
TEE: transesophageal echocardiography
WACA: wide antral circumferential ablation

## Clinical Perspective

### What Is Known

- Pulsed field ablation (PFA) is a novel ablation modality for treating atrial fibrillation based on irreversible electroporation of cardiac tissue.
- The current standard for PFA using a pentaspline catheter is eight applications per pulmonary vein (PV), with four in basket and four in flower configuration.

### What the Study Adds

- This study is the first to compare the efficacy of pulmonary vein isolation using a novel pentaspline PFA protocol (16 applications per PV) with the standard pentaspline PFA protocol (8 applications per PV).
- The novel pentaspline PFA protocol was associated with significantly higher freedom from atrial arrhythmia at 1 year compared with the standard pentaspline PFA protocol (73.9% vs. 62.3%; *p* < 0.05).
- Patients with persistent atrial fibrillation may benefit more from the novel protocol (66.1% vs. 45.3%; *p* < 0.05) than patients with paroxysmal atrial fibrillation (78.8% vs. 75.7%; *p* = ns).
- Serious adverse events were comparable in both groups (*p* = ns).

## 1 Introduction

Pulmonary vein isolation (PVI) is an established treatment option for both paroxysmal atrial fibrillation (PAF) and persistent atrial fibrillation (PersAF). Thermal ablation techniques such as cryoballoon ablation (CBA) and radiofrequency ablation (RFA) are considered standard procedures for performing PVI [1, 2].

Pulsed field ablation (PFA) has been introduced as a non‐thermal ablation modality, causing irreversible damage of cardiac tissue through the application of high‐amplitude electrical pulses [3, 4]. PFA with the pentaspline catheter may offer a more favorable safety profile than thermal ablation, as the risk of damage to extracardiac tissue is lower [5, 6] while offering efficacy comparable to CBA and RFA [7, 8, 9, 10].

The current standard for PFA with the pentaspline catheter (FARAWAVE, Boston Scientific Corp., Marlborough, MA) is eight applications (four in basket and four in flower configuration) per pulmonary vein (PV) [11]. Other ablation protocols with a reduced number of four PFA applications per PV [12] or additional two PFA applications per PV using a different catheter configuration [13] showed no significant improvement in the rate of atrial arrhythmia (AA) after ablation compared to the standard protocol.

The efficacy of standard pentaspline PFA using an increased number of applications per PV has not yet been investigated in patients undergoing PVI for atrial fibrillation (AF). We therefore conducted a prospective study to evaluate the efficacy, i.e., freedom from AA after 1 year, and safety of using 8 additional applications per PV compared with the standard pentaspline PFA protocol.

## 2 Methods

### 2.1 Study Design

This single‐center study was prospective, open‐label, and non‐randomized to evaluate the efficacy of additional PFA applications in patients undergoing PVI with a pentaspline catheter. A total of 292 consecutive patients with PAF or PersAF who underwent PFA‐PVI at University Hospital Düsseldorf, Düsseldorf, Germany, were included.

The first 50 patients treated with pentaspline PFA were excluded from the analysis to account for the learning curve. Additionally, patients with a history of PVI or those requiring additional ablation beyond PVI were excluded. Patients were divided into two groups based on the number of applications delivered per PV. From January to November 2023, patients were treated with 16 applications per PV (group PFA‐16; 6 x basket configuration, 10x flower configuration). Subsequently, from December 2023 to June 2024, patients were treated with 8 applications per PV (group PFA‐8; 4 x basket configuration, 4x flower configuration).

The study was approved by the Ethics Committee of the University of Düsseldorf (number 2019‐555_5) and complies with the Declaration of Helsinki. All study participants provided their written consent.

### 2.2 Procedure

The procedural techniques and methods for pentaspline PFA have been described in detail previously [14, 15]. On the day of ablation, oral anticoagulation was discontinued until evening in accordance with the minimally interrupted anticoagulation strategy [16]. All procedures were performed under deep sedation using propofol, midazolam and, if appropriate, opiates. Left atrial thrombus was excluded by transesophageal echocardiography (TEE). Femoral vein access was performed under ultrasound guidance. An octopolar steerable catheter (Inquiry, Abbott Medical, Green Oaks, IL, USA) was subsequently placed in the coronary sinus. After administration of heparin (target clotting time 300–350 s), a single transseptal puncture was performed under TEE guidance using the FARADRIVE sheath (Boston Scientific Corp., Marlborough, MA). The pentaspline PFA catheter (FARAWAVE 31 mm, Boston Scientific Corp., Marlborough, MA) was inserted into the left atrium through the FARADRIVE sheath, and PV angiography was performed in the standard projections in all patients. At each PV, four applications were performed in basket configuration and four in flower configuration (group PFA‐8) or six applications in basket configuration and ten in flower configuration (group PFA‐16). This approach was chosen to ensure optimal coverage of the PV antrum. In both groups, the pentaspline PFA catheter was rotated by 36° after each pair of applications. Adequate catheter‐tissue contact was assessed by the operator through fluoroscopically visible indentation of the splines and by haptic feedback. PVI was defined as a complete elimination of PV signals and assessment of exit block.

Periprocedural management, including anesthesia strategy, anticoagulation regimen, vascular access, and postprocedural care, was standardized and remained unchanged throughout the entire study period.

### 2.3 Follow‐Up

Immediately after the procedure, patients were monitored by ECG telemetry for at least 48 h. Follow‐up visits including a 12‐lead ECG and a 48‐h Holter ECG were scheduled at 3 and 12 months after the procedure. Outside of scheduled follow‐up visits, a 12‐lead ECG and a 24‐h Holter ECG were performed in the outpatient clinic when symptoms recurred.

The primary endpoint was defined as freedom from AA, i.e., atrial fibrillation, atrial flutter, or atrial tachycardia, after a 90‐day blanking period. Recurrence was defined as any documented AA episode lasting > 30 s, confirmed by a 12‐lead ECG or a Holter ECG. Antiarrhythmic drugs (AAD) were discontinued after the blanking period per protocol; any use of AAD post‐blanking or repeat ablation during the blanking period was considered a recurrence.

Secondary endpoints comprised procedural characteristics, changes in laboratory values (hemoglobin, serum creatinine, and eGFR), changes in myocardial injury markers (creatine kinase and high‐sensitivity cardiac troponin T), and serious adverse events (cardiac tamponade, bleeding necessitating transfusion, pneumonia, stroke or transient ischemic attack, phrenic nerve palsy, death).

### 2.4 Measurement of Hemolysis Parameters

For measurement of the parameters hemoglobin (Hb) and serum creatinine (SCr), blood samples were taken from the peripheral vein before ablation (T0) and on the first day after ablation (T2). Hb levels were determined using the Sysmex XN‐9000 analyzer (Sysmex Corp., Kobe, Japan). The reference range for Hb was 11.9–14.6 g/dL for women and 13.5–16.9 g/dL for men. SCr levels were determined using a standardized UV kinetic assay with photometric detection on the Cobas 8000 analyzer, module C701 (Roche Diagnostics, Basel, Switzerland). The reference range for SCr was < 0.9 mg/dL for women and < 1.2 mg/dL for men. For the calculation of the estimated glomerular filtration rate (eGFR), the Chronic Kidney Disease Epidemiology Collaboration (CKD‐EPI) equation was used [17]. AKI was defined and staged according to the Kidney Disease: Improving Global Outcomes (KDIGO) guidelines [18].

### 2.5 Measurement of Myocardial Injury Markers

To determine the parameters creatine kinase (CK) and high‐sensitivity cardiac troponin T (hs‐cTnT), blood samples were taken from the coronary sinus before ablation (T0), immediately after ablation (T1), and from the peripheral vein on the first day after ablation (T2). CK and hs‐cTnT levels were quantified using tests from Roche Diagnostics (Basel, Switzerland). CK levels were determined using a standardized UV kinetic assay with photometric detection on the Cobas 8000 analyzer, module C701. The reference range for CK was ≤ 145 U/L for women and ≤ 171 U/L for men [19]. Hs‐cTnT was measured using the Elecsys immunoassay, which has a coefficient of variation of ≤ 10% at the upper reference limit of 99% (14 ng/L) [20].

### 2.6 Statistical Analysis

Sample size estimation was based on the anticipated difference in freedom from AA at 12 months between two groups receiving different numbers of PFA applications per PV with the pentaspline catheter. Assuming a moderate effect size of approximately 15 percentage points [21], with a two‐sided alpha of 0.05 and a desired power of 80%, a total sample size of slightly more than 290 patients was considered sufficient to detect a clinically meaningful difference between groups. The assumed effect size of 15% was based on previously published clinical data from prospective PFA studies, including the inspIRE trial [21], which demonstrated clinically meaningful differences in AA freedom depending on lesion delivery strategy. Given the emerging nature of PFA technology, this difference was considered a reasonable and conservative estimate for sample size calculation.

Statistical analyses were performed using IBM SPSS Statistics 30 (IBM Corp., Armonk, NY, USA) and GraphPad Prism 10.3.1 (GraphPad Software Inc., Boston, MA, USA). Continuous variables were expressed as mean ± SD or median with interquartile ranges (IQR), as appropriate. Normal distribution was assessed using the Kolmogorov–Smirnov test. The unpaired *t*‐test or Mann–Whitney *U* test was used for comparison of continuous variables), as appropriate. Nominal parameters were presented as absolute numbers and percentages and compared using Pearson's chi‐squared test. Arrhythmia‐free survival was estimated using Kaplan–Meier curves, and comparisons between groups were made using the log‐rank test. A multivariable Cox proportional hazards regression model adjusted for baseline covariates was used to evaluate predictors of arrhythmia‐free survival. Laboratory parameters were analyzed with the Friedman test. A *p*‐value < 0.05 was considered significant.

## 3 Results

**Figure 1:**
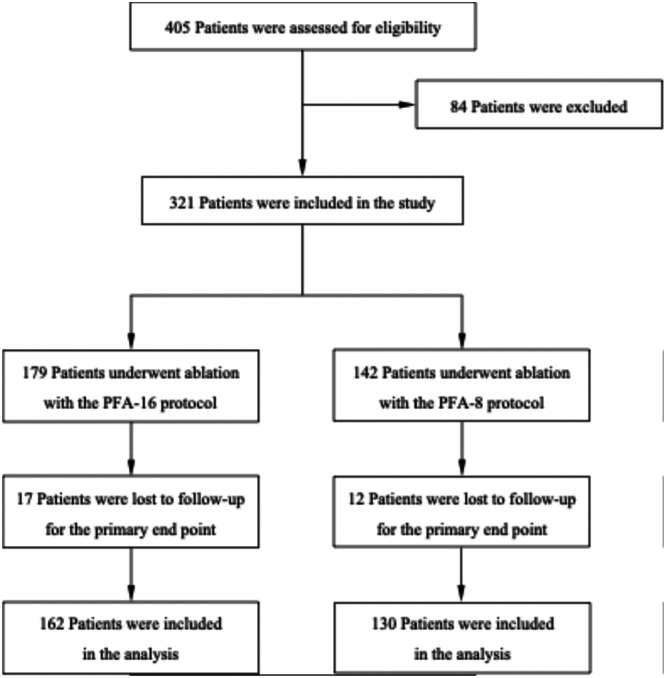
Study flow chart.

A total of 292 patients (median age: 70 [61–77] years; 58% male) were included in the study, 130 patients in group PFA‐8 and 162 patients in group PFA‐16, respectively. An overview of the participant inclusion process is provided in the study flow chart (Figure 1).

Overall, 174 patients (60%) had PAF, whereas the remaining 118 patients (40%) had PersAF. Of the patients with PersAF, one patient (1%) in group PFA‐8 and seven patients (4%) in group PFA‐16 had long‐term PersAF (*p *= ns). There were no significant differences between the two groups (Table 1). Median left atrial volume index (LAVI) was significantly higher in PersAF compared with PAF (39 [32–49] mL/m² vs. 32 [25–42] ml/m²; *p* < 0.001).

**Table 1:** Baseline characteristics.

Baseline characteristics	Overall (*n* = 292)	PFA‐8 (*n* = 130)	PFA‐16 (*n* = 162)	*p*‐value
Age, years	70 (61–77)	71 (62–76)	69 (61–77)	0.473
Sex (male), %	169 (58%)	69 (53%)	100 (62%)	0.137
BMI, kg/m²	28 (24–32)	28 (24–32)	27 (24–32)	0.751
CHA₂DS₂‐VASc score	3 (2–4)	3 (2–4)	3 (2–4)	0.256
LAVI, ml/m²	35 (28–44)	35 (25–41)	37 (30–44)	0.053
Type of atrial fibrillation, %				
Paroxysmal	174 (60%)	74 (57%)	100 (62%)	0.406
Persistent	118 (40%)	56 (43%)	62 (38%)	0.443
History of AF before ablation, days	349 (97–1017)	361 (132–691)	332 (81–1127)	0.993
LVEF, %				
Normal	233 (80%)	103 (79%)	130 (80%)	0.830
Mildly reduced	13 (4%)	5 (4%)	8 (5%)	0.653
Moderately reduced	27 (9%)	13 (10%)	14 (9%)	0.691
Severely reduced	19 (7%)	9 (7%)	10 (6%)	0.796
Heart failure, %	107 (37%)	50 (38%)	57 (35%)	0.564
Hypertension, %	178 (61%)	81 (62%)	97 (60%)	0.672
Diabetes mellitus, %	48 (16%)	18 (14%)	30 (19%)	0.284
Coronary artery disease, %	99 (34%)	40 (31%)	59 (36%)	0.311
History of stroke or TIA, %	29 (10%)	13 (10%)	16 (10%)	0.972
Obstructive sleep apnea, %	36 (12%)	12 (9%)	24 (15%)	0.149
eGFR, mL/min	70 ± 21	70 ± 19	70 ± 22	0.971
Beta blockers, %	257 (88%)	116 (89%)	141 (87%)	0.566
Antiarrhythmic drugs, %				
Class I	22 (8%)	6 (5%)	16 (10%)	0.090
Class III	26 (9%)	11 (8%)	15 (9%)	0.812

Abbreviations: AF, atrial fibrillation; BMI, body mass index; eGFR, estimated glomerular filtration rate; LAVI, left atrial volume index; LVEF, left ventricular ejection fraction; TIA, transient ischemic attack.

### 3.1 Primary Endpoint: Freedom From AA

**Figure 2:**
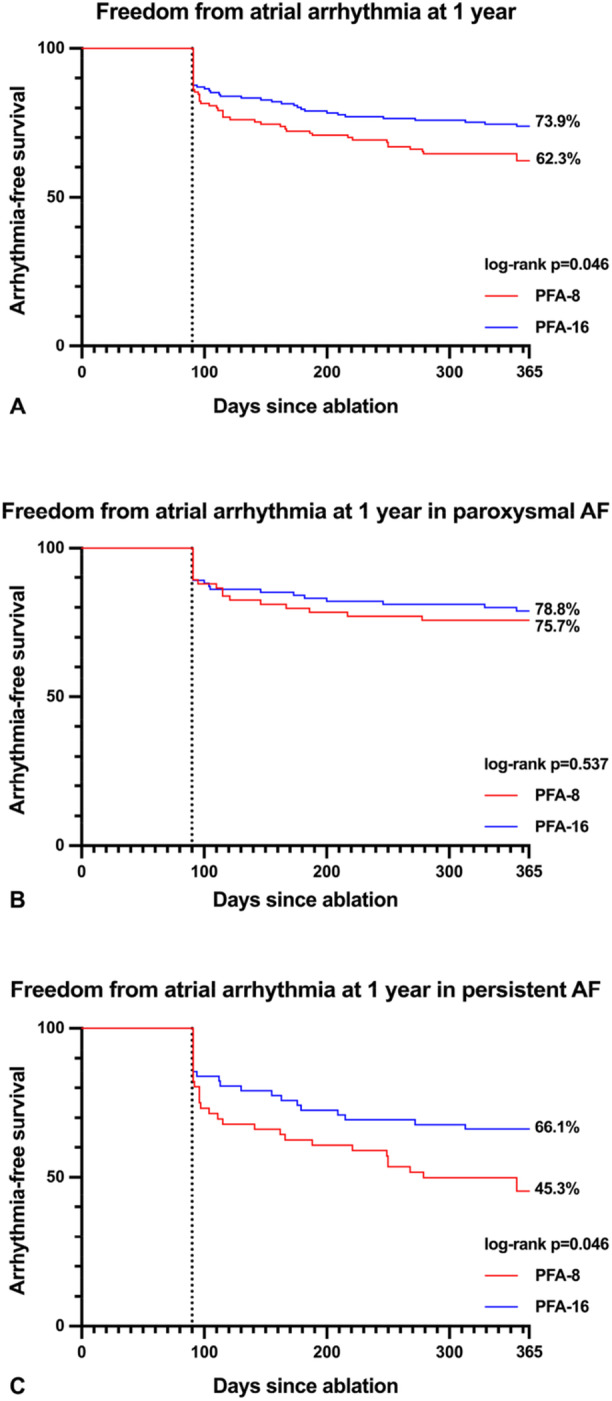
Freedom from atrial arrhythmia at 1 year. Kaplan‐Meier analysis of freedom from atrial arrhythmia in group PFA‐8 and group PFA‐16: (A) All patients, (B) Patients with paroxysmal atrial fibrillation, (C) Patients with persistent atrial fibrillation. *AF* atrial fibrillation.

The median follow‐up period was 371 (341–465) days. At 12 months, the Kaplan‐Meier estimate of freedom from AA was significantly higher in the PFA‐16 group compared with the PFA‐8 group (73.9% vs. 62.3%; log‐rank *p* < 0.05) (Figure 2).

Subgroup analysis showed differing outcomes between patients with PAF and those with PersAF. Patients with PAF had a comparable freedom from AA (75.7% in group PFA‐8 vs. 78.8% in group PFA‐16; *p* = ns), whereas in patients with PersAF, freedom from AA was significantly higher in group PFA‐16 (66.1% vs. 45.3% in group PFA‐8; *p* < 0.05) (Figure 2).

Multivariable Cox proportional hazards regression adjusted for baseline covariates (age, sex, LAVI, type of AF, and history of AF) showed that none of these covariates were significantly associated with arrhythmia‐free survival (*p *= ns) (Supporting Information S1, Table S1).

### 3.2 Secondary Endpoint: Procedural Characteristics

Complete PVI was achieved in all patients. The median procedure time was significantly shorter in group PFA‐8 (44 [37–52] minutes) compared with group PFA‐16 (58 [50–72] minutes; *p* < 0.001). Both median fluoroscopy time and fluoroscopy dose were significantly lower in group PFA‐8 than in group PFA‐16. The procedural characteristics are summarized in Table 2.

**Table 2:** Procedural characteristics.

Procedural data	Overall (*n* = 292)	PFA‐8 (*n* = 130)	PFA‐16 (*n* = 162)	*p*‐value
Procedure time, min	52 (44–67)	44 (37–52)	58 (50–72)	< 0.001
Fluoroscopy time, min	15 (12–19)	12 (10–15)	17 (14–22)	< 0.001
Fluoroscopy dose, cGy×cm²	553 (380–777)	498 (369–684)	608 (425–904)	0.005

A total of eight serious adverse events (2.7%) were observed, with four events (3.1%) in group PFA‐8 and four events (2.5%) in group PFA‐16. In the PFA‐8 group, serious adverse events included cardiac tamponade (*n* = 1 [0.8%]) and pneumonia (*n* = 1 [0.8%]). Other adverse events (*n* = 2 [1.5%]) included third‐degree atrioventricular block requiring permanent pacemaker implantation (*n* = 1 [0.8%]) and femoral nerve injury (*n* = 1 [0.8%]). In group PFA‐16, serious adverse events included cardiac tamponade (*n* = 1 [0.6%]), major bleeding (*n* = 1 [0.6%]), pneumonia (*n* = 1 [0.6%]), and a transient ischemic attack (*n* = 1 [0.6%]). No cases of stroke, phrenic nerve palsy, or death occurred. The rate of serious adverse events did not differ significantly between both groups (Table 3).

**Table 3:** Safety endpoints.

Safety endpoints	Overall (*n* = 292)	PFA‐8 (*n* = 130)	PFA‐16 (*n* = 162)	*p*‐value
Cardiac tamponade	2 (0.7%)	1 (0.8%)	1 (0.6%)	0.876
Major bleeding	1 (0.3%)	0 (0%)	1 (0.6%)	0.370
Pneumonia	2 (0.7%)	1 (0.8%)	1 (0.6%)	0.876
Stroke or TIA	1 (0.3%)	0 (0%)	1 (0.6%)	0.370
Phrenic nerve palsy	0 (0%)	0 (0%)	0 (0%)	—
Death	0 (0%)	0 (0%)	0 (0%)	—
Other	2 (0.7%)	2 (1.5%)	0 (0%)	0.113
Total	8 (2.7%)	4 (3.1%)	4 (2.5%)	0.752

Abbreviation: TIA, transient ischemic attack.

### 3.3 Secondary Endpoint: Hemolysis Parameters

**Figure 3:**
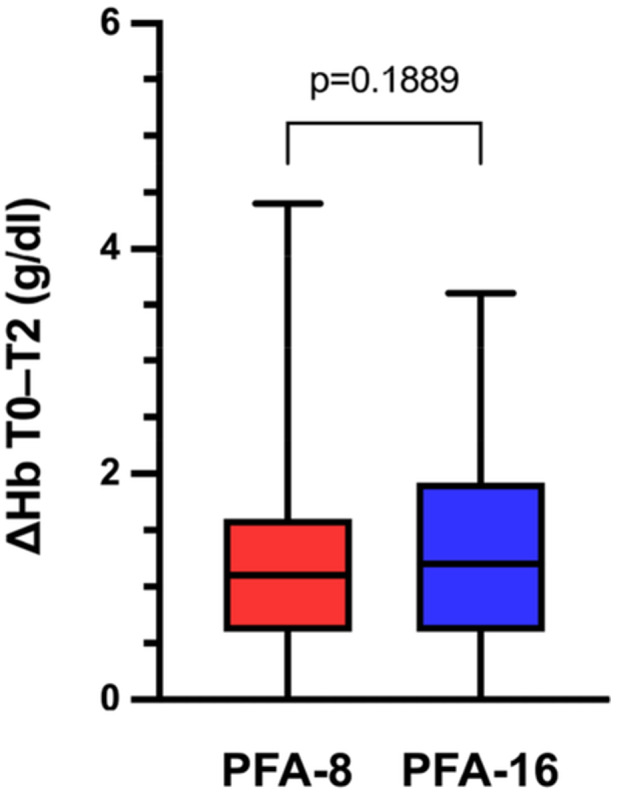
Differences of hemoglobin before and after ablation in group PFA‐8 and group PFA‐16. ΔHb (T0 to T2). Values are presented as median with minimum and maximum ranges. *Hb* hemoglobin; *T0* pre‐ablation; *T2* first day post‐ablation.

There were no significant differences in baseline Hb, SCr, or eGFR between the two groups. Hb levels decreased significantly in both groups (*p* < 0.0001) (Table 4). However, the delta values did not differ significantly between the two groups (Figure 3). There were no significant changes in SCr or eGFR levels compared with baseline in either group, or significant differences between the two groups. A total of seven instances (3.0%) of an SCr increase consistent with stage 1 AKI were observed, with no differences between the two groups. No cases of stage 2 or stage 3 AKI were observed (Table 5). In 4 of these instances, baseline SCr levels were elevated (Supporting Information, Table S2).

**Table 4:** Changes in hemoglobin.

Changes in hemoglobin	Overall (*n* = 292)	PFA‐8 (*n* = 130)	PFA‐16 (*n* = 162)	*p*‐value
Hemoglobin (T0), g/dL	14.14 ± 1.59	14.16 ± 1.46	14.13 ± 1.68	0.887
Hemoglobin (T2), g/dL	12.94 ± 1.62	13.05 ± 1.57	12.86 ± 1.66	0.376
*p*‐value		< 0.0001	< 0.0001	
ΔHemoglobin 1–2, %	101 (43.3%)	39 (41.1%)	62 (44.9%)	0.558
ΔHemoglobin 2.1–3, %	30 (12.9%)	8 (8.4%)	22 (15.9%)	0.092
ΔHemoglobin > 3, %	8 (3.4%)	3 (3.2%)	5 (3.6%)	0.848

*Note: T0* pre‐ablation; *T2* first day post‐ablation.

**Table 5:** Changes in renal function.

Changes in renal function	Overall (*n* = 292)	PFA‐8 (*n* = 130)	PFA‐16 (*n* = 162)	*p*‐value
Serum creatinine (T0), mg/dL	0.99 (0.81–1.20)	0.96 (0.78–1.18)	1.01 (0.83–1.22)	0.199
Serum creatinine (T2), mg/dL	0.99 (0.84–1.18)	0.99 (0.81–1.23)	1.01 (0.85–1.19)	0.370
*p*‐value		0.574	0.828	
eGFR (T0), mL/min	70 ± 21	70 ± 19	70 ± 22	0.974
eGFR (T2), mL/min	70 ± 21	69 ± 20	70 ± 22	0.715
*p*‐value		0.235	0.864	
KDIGO AKI stage 1, %	7 (3.0%)	4 (4.2%)	3 (2.2%)	0.371
KDIGO AKI stage 2, %	0 (0%)	0 (0%)	0 (0%)	—
KDIGO AKI stage 3, %	0 (0%)	0 (0%)	0 (0%)	—

Abbreviations:AKI, acute kidney injury; eGFR, estimated glomerular filtration rate; KDIGO, Kidney Disease: Improving Global Outcomes; *T0*, pre‐ablation; *T2*, first day post‐ablation.

### 3.4 Secondary Endpoint: Myocardial Injury Markers

**Figure 4:**
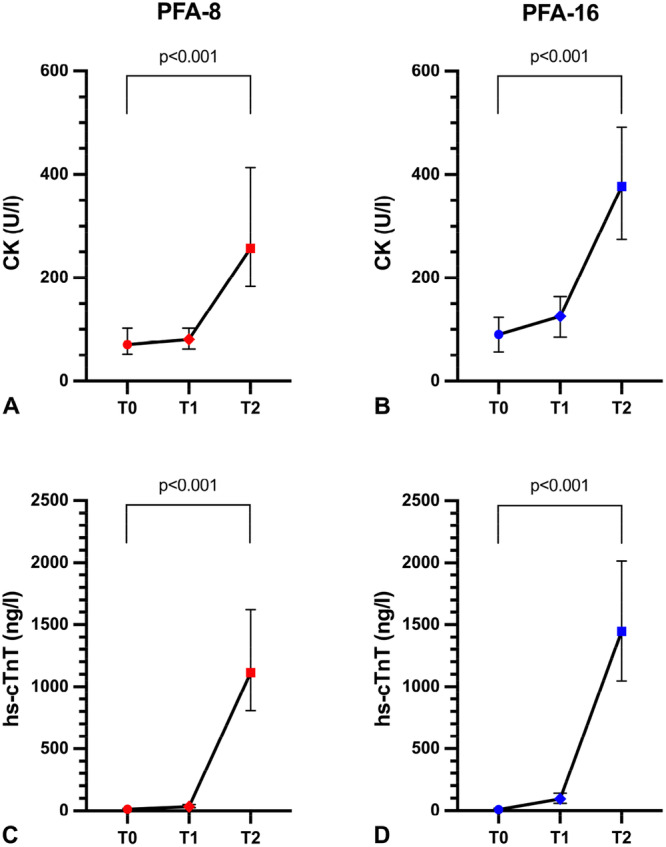
Changes in myocardial injury markers. CK and hs‐cTnT levels were measured before ablation (T0), on the same day after ablation (T1), and on the first day post‐ablation (T2): (A) CK levels in group PFA‐8, (B) CK levels in group PFA‐16, (C) hs‐cTnT levels in group PFA‐8, (D) hs‐cTnT levels in group PFA‐16. Values are presented as median with interquartile ranges. *CK* creatine kinase; *hs‐cTnT* high‐sensitivity cardiac troponin T.

**Figure 5:**
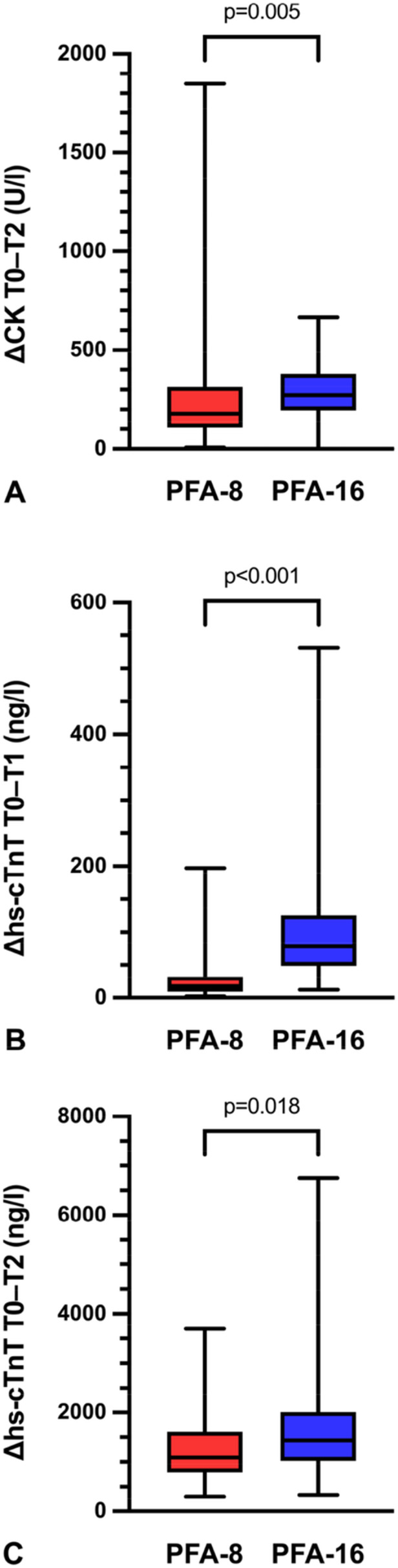
Differences of myocardial injury markers before and after ablation in group PFA‐8 and group PFA‐16. (A) ΔCK (T0 to T2), (B) Δhs‐cTnT (T0 to T1), (C) Δhs‐cTnT (T0 to T2). Values are presented as median with minimum and maximum ranges. *CK* creatine kinase; *hs‐cTnT* high‐sensitivity cardiac troponin T; *T0* pre‐ablation; *T1* same day post‐ablation; *T2* first day post‐ablation.

Compared with baseline values, both CK and hs‐cTnT levels increased significantly in both groups (*p* < 0.001) (Figure 4). However, the increases were more pronounced in group PFA‐16, resulting in significantly higher delta values for both parameters compared with group PFA‐8 (*p* < 0.05) (Figure 5).

## 4 Discussion

This study is the first to compare the efficacy of PFA using 16 applications per PV (6 x basket, 10 x flower) with the standard PFA protocol (4 x basket, 4 x flower) in patients undergoing PVI with the pentaspline catheter.

The main findings of our study are that pentaspline PFA with 16 applications per PV significantly improved freedom from AA at 1 year compared with standard pentaspline PFA, both in the overall cohort (73.9% vs. 62.3%; *p* < 0.05) and in the subgroup of patients with PersAF (66.1% vs. 45.3%; *p* < 0.05). Notably, the rate of serious adverse events did not differ significantly between the two groups. Changes reflecting hemolysis (Hb, SCr, and eGFR), as well as the incidence of AKI, did not differ significantly between the two groups. As expected, pentaspline PFA using 16 applications per PV was associated with a significantly greater increase in myocardial injury markers (CK and hs‐cTnT).

### 4.1 Potential Benefits of Additional PFA Applications

Antral fibrosis is a recognized contributor to the maintenance of atrial fibrillation [22]. The DECAAF II study [22] demonstrated that patients with PersAF exhibit increased fibrosis in the PV region. A meta‐analysis by Proietti et al. [23] suggested that RFA‐PVI using wide antral circumferential ablation (WACA) is associated with improved long‐term rhythm control compared to RFA‐PVI with ostial ablation.

Our data suggest that sustained antral ablation may be more difficult to achieve in PersAF due to larger atria and wider antra, and that patients with PersAF may benefit from additional applications to achieve circumferential coverage. However, it can be speculated that anatomy‐guided lesion delivery may achieve comparable results.

The present study was based on the hypothesis that PVI with eight additional PFA applications, including six in the flower configuration, may improve clinical outcome by extending lesions into the surrounding antral myocardium, thereby more effectively targeting antral fibrosis. Accordingly, extended lesion formation may be reflected by the pronounced increase of CK and hs‐cTnT in the PFA‐16 group.

Tilz et al. [24] demonstrated that the addition of 2 PFA applications in the flower configuration (“WACA‐PFA”) resulted in significantly larger lesion areas compared with the conventional pentaspline PFA. At 1 year, arrhythmia‐free survival was numerically higher in patients treated with WACA‐PFA compared to standard pentaspline PFA (94% vs. 87%; *p* = 0.68). This difference was not statistically significant, which may be attributed to the small sample size (*n* = 30; PersAF 33%). In contrast, our study included a total of 292 patients, of whom 118 had PersAF.

Recently, Schaack et al. [13] introduced a novel pentaspline PFA protocol including two additional applications in a modified basket configuration referred to as the olive configuration. Although the olive strategy showed no significant improvement in freedom from AA compared with the standard pentaspline PFA procedure, it might be associated with a lower rate of reconnected PVs. The main difference between our study and the olive strategy lies in the ablation approach, while Schaack et al. performed ostial ablation, our strategy involved antral ablation.

Badertscher et al. [12] have shown that pentaspline PFA using a reduced number of 4 applications per PV (2 x basket, 2 x flower) may be as efficient and safe as the standard pentaspline PFA protocol. The inclusion of two additional applications at the right‐sided carina in all patients may limit the comparability of this study to both our study and the olive study. Moreover, the study did not present a subgroup analysis investigating outcomes in patients with PersAF, who may benefit the most from an extended ablation protocol.

### 4.2 Safety

Our study shows that pentaspline PFA‐PVI with 16 applications per PV is a safe procedure. There was a total of eight serious adverse events (2.7%), with no significant differences between both groups. The absence of phrenic nerve palsy in our study is consistent with the high tissue specificity of pentaspline PFA [3, 4]. However, recent studies have reported hemolysis as a pentaspline PFA‐related adverse event, with the severity likely being dose‐dependent [25, 26, 27].

There were concerns that pentaspline PFA could cause significant hemolysis in patients undergoing PVI. However, the incidence of hemolysis‐associated significant anemia was low, suggesting that the clinical consequences of hemolysis during pentaspline PFA are usually minor and possibly dose‐dependent [25, 26, 27]. The MANIFEST‐17K study showed that hemolysis‐related acute kidney injury (AKI) was very rare (0.03%) and may be related to the number of PFA applications (≥ 96 applications per patient) [28].

In a previous study of our institution, pentaspline PFA using up to 16 applications per PV was associated with a significant increase in hemolysis markers. However, no cases of AKI were reported [29]. Notably, hemolysis‐related AKI has been reported predominantly after pentaspline PFA with an extensive number of applications (≥ 100 applications) [26, 28] which exceeds the number of applications used in our study (64 applications per procedure). In addition to the previous study, we analyzed changes in Hb, SCr, and eGFR from baseline in this study cohort. These changes did not differ significantly between the two groups. In seven patients, an increase in SCr consistent with stage 1 AKI was observed, with no differences between both groups. No cases of higher‐grade AKI were observed. Elevated baseline SCr levels and decreased baseline GFR may be associated with an increased risk of developing AKI [28, 30]. In this study, baseline SCr was elevated in four of the seven patients, including two patients with chronic kidney disease.

### 4.3 Limitations

The study was conducted in a non‐randomized, single‐center setting, which may have introduced potential bias related to patient selection, temporal effects, and institution‐specific procedural protocols.

Furthermore, the absence of a standardized PVI confirmation protocol with systematic assessment of entrance block, exit block, and electroanatomical mapping represents a limitation and should be considered when interpreting the findings.

Assessment of AA recurrence relied on intermittent Holter ECG monitoring rather than continuous rhythm monitoring with implantable loop recorders, which may underestimate the true burden of recurrence, especially for asymptomatic or short‐lasting episodes. Although follow‐up was conducted in line with guideline‐based recommendations, the absence of blinding may have introduced observational bias. Further randomized studies with continuous ECG monitoring may be necessary to confirm these results.

## 5 Conclusion

This study demonstrates that a novel pentaspline PFA protocol using 16 applications per PV offers superior efficacy compared with the standard pentaspline PFA protocol (8 applications per PV), particularly in patients with PersAF. The improved clinical outcome was not associated with an increase in serious adverse events. These findings suggest that the use of additional pentaspline PFA applications may be associated with an improved outcome in patients with PersAF undergoing PVI. Further studies using 3D mapping may be necessary to further evaluate the role of higher dose delivery in PersAF.

## Conflicts of Interest

The authors declare no conflicts of interest.

## Supporting information


**Table S1:** Multivariable Cox proportional hazards regression adjusted for baseline covariates. **Table S2:** Case descriptions of acute kidney injury.

## Data Availability

All relevant data are within the manuscript.
