# Supplementary material for: Impact of Pulsed Field Ablation Dosing on Outcome After Pulmonary Vein Isolation Using a Pentaspline Ablation Catheter: A Prospective Comparison of 8 Versus 16 Applications
Source: J Cardiovasc Electrophysiol. 2026 Jan 20;37(3):583–92. doi: 10.1111/jce.70266 (PMC12980466; doi:10.1111/jce.70266)
Supplement: Supplementary file 1 — Table S1: Multivariable Cox proportional hazards regression adjusted for baseline covariates. Table S2: Case descriptions of acute kidney injury. [file JCE-37-583-s001.docx]

**Supplementary material**

**Table S1.** Multivariable Cox proportional hazards regression adjusted for baseline covariates

| **Covariate** | ***p*-value** |
| --- | --- |
| Age | 0.556 |
| Sex | 0.804 |
| LAVI | 0.197 |
| Type of atrial fibrillation | 0.299 |
| History of atrial fibrillation | 0.268 |

*LAVI* left atrial volume index.

**Table S2.** Case descriptions of acute kidney injury

|  | Group | SCr at baseline | KDIGO AKI Stage | Description |
| --- | --- | --- | --- | --- |
| Patient 1 | PFA-16 | 1.61 mg/dl | Stage 1 | An 84-year-old female patient with a history of stage 3 CKD and hypertension underwent ablation for persistent AF. The patient’s hospital stay was prolonged due to AKI and major bleeding. The patient was discharged in stable condition on post-ablation day 12 with a SCr below baseline at 1.48 mg/dl. |
| Patient 2 | PFA-16 | 1.26 mg/dl | Stage 1 | A 72-year-old male patient with a history of HFrEF and coronary artery disease underwent ablation for persistent AF. Post-ablation, fluid intake was low. On post-ablation day 1, SCr was 2.06 mg/dl. Renal function recovered after mild volume replacement, with a SCr of 1.39 mg/dl on post-ablation day 2. The patient was discharged in stable condition on post-ablation day 3. |
| Patient 3 | PFA-8 | 0.87 mg/dl | Stage 1 | A 69-year-old male patient with a history of hypertension underwent ablation for paroxysmal AF. On post-ablation day 1, the patient was discharged in stable condition with a SCr of 1.31 mg/dl. Renal function had recovered by the 3-month follow-up. |
| Patient 4 | PFA-8 | 2.08 mg/dl | Stage 1 | A 58-year-old male patient with a history of HFrEF and coronary artery disease underwent ablation for paroxysmal AF. On post-ablation day 1, SCr was 2.91 mg/dl. Xipamide was discontinued, the dose of torasemide was reduced, and volume replacement was initiated. On post-ablation day 3, SCr was below baseline at 1.81 mg/dl. The patient requested premature discharge on post-ablation day 4 against medical advice. Renal function had recovered by the 3-month follow-up. |
| Patient 5 | PFA-8 | 0.71 mg/dl | Stage 1 | A 65-year-old female patient with a history of HFrEF and stroke underwent ablation for paroxysmal AF. On post-ablation day 1, SCr was 1.09 mg/dl. The patient was discharged in stable condition on post-ablation day 2. Renal function had recovered by the 3-month follow-up. |
| Patient 6 | PFA-8 | 0.69 mg/dl | Stage 1 | A 71-year-old male patient with a history of HFrEF, hypertension, and diabetes underwent ablation for paroxysmal AF. On post-ablation day 1, SCr was 1.17 mg/dl. The patient was discharged in stable condition on post-ablation day 2. Renal function had recovered by the 3-month follow-up. |
| Patient 7 | PFA-16 | 1.60 mg/dl | Stage 1 | A 71-year-old male patient with a history of stage 3 CKD, HFrEF, and coronary artery disease underwent ablation for persistent AF. On post-ablation day 1, SCr was 1.90 mg/dl, and on post-ablation day 4, SCr peaked at 2.16 mg/dl. The patient was discharged in stable condition on post-ablation day 5. |

*AF* atrial fibrillation; *AKI* acute kidney injury; *CKD* chronic kidney disease; *SCr* serum creatinine; *HFrEF* heart failure with reduced ejection fraction; *KDIGO* Kidney Disease: Improving Global Outcomes.
